# Identification of plastid genomic regions inferring species identity from *de novo* plastid genome assembly of 14 Korean-native *Iris* species (*Iridaceae*)

**DOI:** 10.1371/journal.pone.0241178

**Published:** 2020-10-26

**Authors:** Yang Jae Kang, Soonok Kim, Jungho Lee, Hyosig Won, Gi-Heum Nam, Myounghai Kwak

**Affiliations:** 1 Division of Bio & Medical Big Data Department (BK4 Program), Gyeongsang National University, Jinju, Republic of Korea; 2 Division of Life Science Department, Gyeongsang National University, Jinju, Republic of Korea; 3 National Institute of Biological Resources, Incheon, Republic of Korea; 4 Green Plant Institute, Yongin, Republic of Korea; 5 Dept. of Biological Science, Daegu University, Gyungsan, Gyungbuk, Republic of Korea; Universita degli Studi della Calabria Dipartimento di Biologia Ecologia e Scienze della Terra, ITALY

## Abstract

*Iris* is one of the largest genera in the family *Iridaceae*, comprising hundreds of species, including numerous economically important horticultural plants used in landscape gardening and herbal medicine. Improved taxonomic classification of *Iris* species, particularly the endangered Korean-native *Iris*, is needed for correct species delineation. To this end, identification of diverse genetic markers from *Iris* genomes would facilitate molecular identification and resolve ambiguous classifications from molecular analyses; however, only two *Iris* plastid genomes, from *Iris gatesii* and *Iris sanguinea*, have been sequenced. Here, we used high-throughput next-generation sequencing, combined with Sanger sequencing, to construct the plastid genomes of 14 Korean-native *Iris* species with one outgroup and predict their gene content. Using these data, combined with previously published plastid genomes from *Iris* and one outgroup (*Sisyrinchium angustifolium*), we constructed a Bayesian phylogenetic tree showing clear speciation among the samples. We further identified sub-genomic regions that have undergone neutral evolution and accurately recapitulate Bayesian-inferred speciation. These contain key markers that could be used to identify and classify *Iris* samples into taxonomic clades. Our results confirm previously reported speciation patterns and resolve questionable relationships within the *Iris* genus. These data also provide a valuable resource for studying genetic diversity and refining phylogenetic relationships between *Iris* species.

## Introduction

The *Iris* genus is comprised of hundreds of species, making it one of the largest in the *Iridaceae* family. This group contains a large number of plants used for aesthetic purposes, such as landscape gardening, as well as many economically important medicinal plants. Portions of *Iris* plants have been used in traditional medicine for detoxification, as well as for treating constipation, stomach ache, and sore throat [1]. *Iris* species are distributed across Europe, Asia, and America, and display high levels of genome diversity and variable ploidy [2–4]. In Korea, several native *Iris* species are distributed across diverse environments, ranging from dry to wet regions. Additionally, some species are currently considered endangered (e.g. *Iris laevigata*, *Iris ruthenica*, and *Iris koreana*) and are subject to legal protection by the Korean government.

To date, the phylogenetic relationships among species in the *Iris* genus have been determined based on genomic regions in the chloroplast and nucleus, such as the internal transcribed spacer (nrITS), *matK*, *ndhF*, *trnL-trnF*, *trnQ-rps16*, and *trnS-trnfM* [5–7]. Although these methods have been used for most members of the *Iridaceae*, it is not clear whether the phylogenetic relationships among clades and closely related species have been clearly identified due to insufficient taxonomic coverage or lack of informative sites [5–7]. Further, in addition to problems arising from insufficient sampling and poor resolution of molecular markers, phylogenetic relationships among species, particularly closely related ones, are often difficult to resolve due to factors such as frequent hybridisation and taxonomic ambiguity [8, 9].

Recently, phylogenetic analysis of whole chloroplast genomes was suggested as an alternative to provide better resolution for species designation [10, 11]. In support of this, consolidation of alignments for the majority of genes in a plastid genome has been successfully used for building species trees in a number of instances [12–14]. Angiosperm speciation, for example, was investigated using plastid genes, providing strong support for the early diverged flowering lineage *Amborella* [12]. *Brassica* speciation was also elucidated with whole-plastid genome sequencing-consolidated plastid gene trees [13]. The development of next-generation sequencing (NGS) technology and advances in bioinformatic tools have further facilitated the assembly of complete plastid genome sequences from plants [12, 15]. However, due to cost and the need for large amounts of computing power, it remains difficult to decipher whole plastid genomes from a sufficient number of samples to elucidate low-level phylogeny and enable delineation of species.

Currently, a total of 280 *Iris* species have been documented in NCBI with taxonomy IDs. However, only two plastid genomes, those from *Iris gatesii* and *Iris sanguinea*, have been sequenced. In this study, we used high-throughput NGS technology, together with Sanger sequencing, to decipher the plastid genomes of 14 Korean-native *Iris* species and predict their gene content. Using these data and the published plastid genomes from *I*. *gatesii* and *I*. *sanguinea*, we compared the *Iris* species by pair-wise Ks value calculation and successfully constructed a Bayesian phylogenetic tree. We then extracted representative regions from whole plastid genomes reflecting the phylogeny of *Iridaceae* using the scores from the neutrality test. The speciation of closely related species was re-verified with traditional phylogenetic analysis using *matK* sequences from 117 *Iris* accessions. From the representative CP genomic regions, the resolution of the *Iris* species classification would be increased for the identification and protection of endangered Korean native *Iris* species.

## Results

### Chloroplast genome sequence assembly from 14 Korean-native *Iris* species

The complete plastid genome sequences were determined for 14 Korean-native *Iris* species and one outgroup species, *Sisyrinchium angustifolium*, using NGS and Sanger sequencing technology (Table 1). Genomic sequences of approximately 0.9–2.3 Gbps were generated from each species using the Illumina platform (Table 2). Plastid genome sequences, ranging from 150,947–153,730 bp in length, were also extracted and assembled. Based on these assembled plastid sequences, 83 genes were predicted for each species (S1 Table). Implementation of a curation process to meet the NCBI submission standard resulted in a total of 63–73 coding genes for each species (Table 3). This variation in the number of coding sequences is partly due to assembly ambiguities (erroneous insertions and variants) that could not be properly translated into start and stop codons for certain genes. These resulted from predicted coding sequences (CDS) that were not in multiples of three or contained an improper codon at the start or end of the protein. Hence, the absence of genes in each assembly does not necessarily indicate the true absence of genes from the evolutionary process. In addition, a total of 30–31 tRNAs and 12 rRNAs were annotated in each plastid assembly. One of three tRNAs, including ’trnG-UCC’, ’trnK-UUU’, and ’trnnull-NNN’, were not annotated in some species, possibly due to sequencing errors. The large single copy (LSC), small single copy (SSC), and inverted repeat (IR) regions were also identified, displaying average lengths of 82,255 bp, 18,060 bp, and 26,053 bp, respectively (Table 3).

**Table 1:** *Iris* species and outgroup used in this study.

Scientific name	Sequencing method	GenBank ID	Reference
*S*. *angustifolium*	Mi-seq	MK593170	In this study
*I*. *sanguinea*	Mi-seq	NC_029227	[28]
*I*. *gatesii*	Hi-seq	NC_024936	[29]
*I*. *laevigata*	Mi-seq	MK593161	In this study
*I*. *setosa*	Mi-seq	MK593168	In this study
*I*. *ensata* var. *spontanea*	Mi-seq	MK593158	In this study
*I*. *pseudoacorus*	Mi-seq	MK593164	In this study
*I*. *lactea* var. *chinensis*	Mi-seq	MK593160	In this study
*I*. *ruthenica*	Hi-seq	MK593167	In this study
*I*. *uniflora* var. *caricina*	Hi-seq	MK593169	In this study
*I*. *domestica*	Mi-seq	MK593156	In this study
*I*. *dichotoma*	Sanger	MK593157	In this study
*I*. *odaesanensis*	Sanger	MK593163	In this study
*I*. *rosii* var. *latifolia*	Mi-seq	MK593166	In this study
*I*. *rosii* var. *rosii*	Mi-seq	MK593165	In this study
*I*. *koreana*	Sanger	MK593159	In this study
*I*. *minutoaurea*	Mi-seq	MK593162	In this study

**Table 2:** NGS sequencing statistics for *Iris* species and outgroup.

Species	Trimmed data (bp)	Percentage of trimmed bases to original raw reads	# of aligned reads to the plastid genome	Plastid genome coverage (x)	Plastid genome length (bp)
*S*. *angustifolium*	1,412,822,734	79.03%	92,374	140	151,305
*I*. *domestica*	2,169,071,850	78.93%	309,614	544	153730
*I*. *ensata* var. *spontanea*	1,305,887,854	71.47%	338,839	661	153,055
*I*. *lactea* var. *chinensis*	1,443,127,793	70.37%	94,059	162	152,294
*I*. *laevigata*	4,340,216,662	86.23%	654,027	381	151,081
*I*. *minutoaurea*	1,133,976,531	69.96%	158,227	276	150,955
*I*. *pseudoacorus*	1,033,295,333	69.29%	143,774	246	152,562
*I*. *rossii* var. *latifolia*	929,066,495	69.65%	122,004	226	152,654
*I*. *rossii* var. *rossii*	1,350,741,033	69.42%	179,282	309	153,083
*I*. *ruthenica*	4,265,652,485	89.30%	2,067,701	1,218	152,287
*I*. *sanguine*	1,330,043,383	70.53%	179,563	309	152,408
*I*. *setose*	1,262,063,831	69.91%	104,833	178	152,921
*I*. *uniflora* var. *caricina*	2,344,526,509	77.33%	256,424	455	152,281

**Table 3:** Summary of plastid genome assemblies and gene annotations.

Species	Plastid genome size	LSC	IRb	SSC	IRa	Predicted genes	High- quality predictions	tRNAs	rRNAs
*S*. *angustifolium*	151305	80790	26177	18161	26177	83	63	30	12
*I*. *dichotoma*	153573	83071	26181	18140	26181	83	71	31	12
*I*. *domestica*	153730	83137	26199	18195	26199	83	70	31	12
*I*. *koreana*	153055	82579	26094	18288	26094	83	68	30	12
*I*. *ensata*	150947	81514	25549	18335	25549	83	72	31	12
*I*. *lactea*	152294	82159	26023	18089	26023	83	72	30	12
*I*. *laevigata*	151081	81144	26022	17893	26022	83	71	31	12
*I*. *minutoaurea*	150955	81516	25549	18341	25549	83	68	30	12
*I*. *odaesanensis*	153620	82777	26784	17275	26784	83	68	31	12
*I*. *pseudoacorus*	152562	82786	25948	17880	25948	83	73	31	12
*I*. *rossii* var. *latifolia*	152654	82212	26183	18076	26183	83	71	31	12
*I*. *rossii* var. *rossii*	153083	82635	26183	18082	26183	83	71	31	12
*I*. *ruthenica*	152287	82311	25920	18136	25920	83	71	31	12
*I*. *setosa*	152921	82900	26067	17887	26067	83	72	31	12
*I*. *uniflora* var. *caricina*	152281	82307	25920	18134	25920	83	71	30	12
Mean (bp)	152423.20	82255.87	26053.27	18060.80	26053.27	83.00	70.13	30.67	12

*LSC: long single copy section, IRa and IRb: inverted repeats, SSC: short single copy section

### *Ks* value-based classification

**Fig 1:**
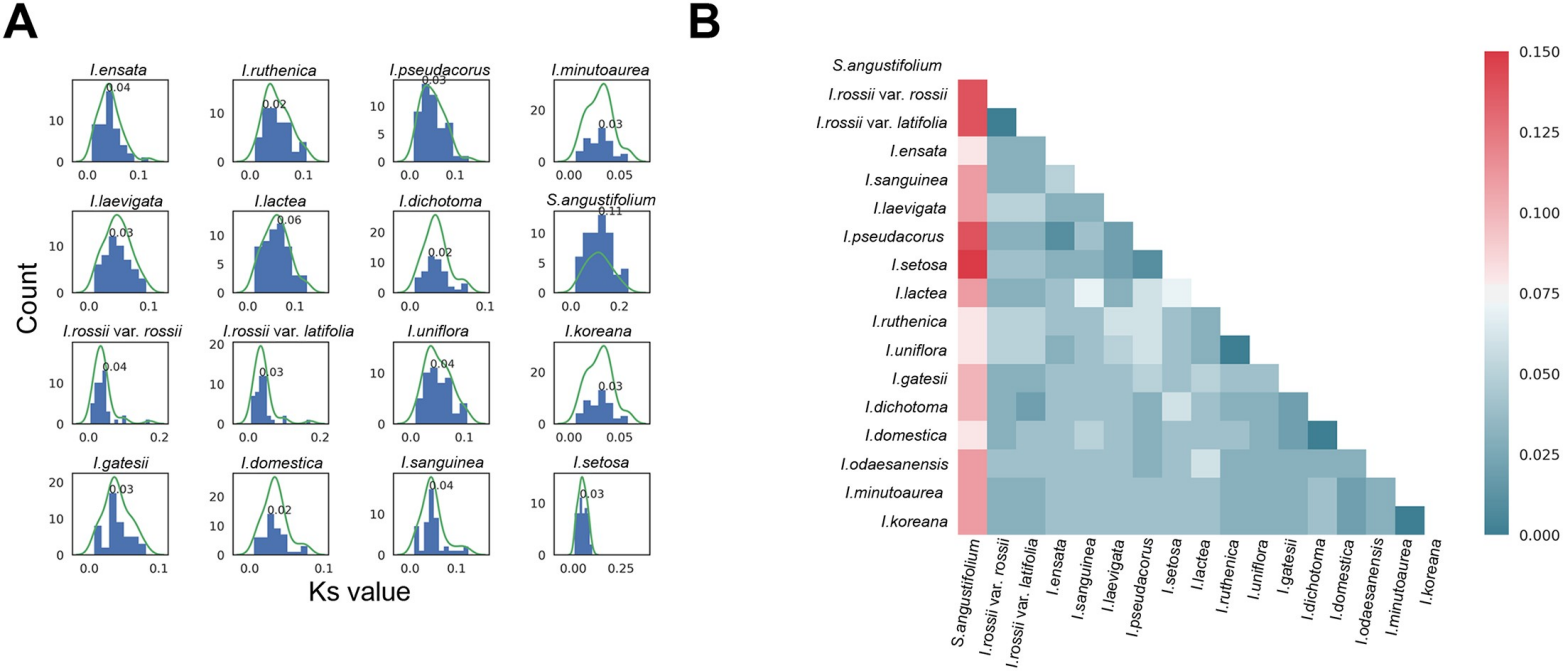
*Ks* distributions for *Iris* species pairwise comparisons. (a) Pairwise *Ks* histogram nested to *I*. *odaesanensis*. (b) All-to-all triangle heatmap for modal values of pairwise *Ks* distributions.

*Ks* values are calculated by estimating synonymous changes within a coding sequence, which are believed to provide a metric for the length of time following speciation, without being affected by the selection process. The pairwise comparison between two species generates a *Ks* value distribution for orthologous gene pairs, and the peak value of the distribution provides a good proxy for estimating relative species divergence time [16]. Therefore, in order to estimate speciation for the 17 *Iris* members in our study, pairwise *Ks* values were calculated. The *Ks* value distributions from *Iris odaesanensis* to each species were then plotted to visualise speciation signals displaying variable peaks (Fig 1a). From all pairwise combinations of *Iris* species and the outgroup (*S*. *angustifolium*), peak *Ks* values were extracted, and we built a triangle distance table of peak *Ks* values (Fig 1b). These data displayed close relationships, such as 1) *I*. *koreana* and *Iris minutoaurea*, 2) *I*. *ruthenica* and *Iris uniflora*, and 3) *Iris rossii* var. *rossii* and *I*. *rossii* var. *latifolia* (Fig 1b). Close relationships were also indicated from a *matK*-based phylogenetic tree generated from a set of 117 Iris accessions displaying the clades: 1) *I*. *koreana* and *I*. *minutoaurea* and 2) *I*. *ruthenica* and *I*. *uniflora* (S1 Fig).

### Species tree reconstruction using the Bayesian method by 57 chloroplast genes

**Fig 2:**
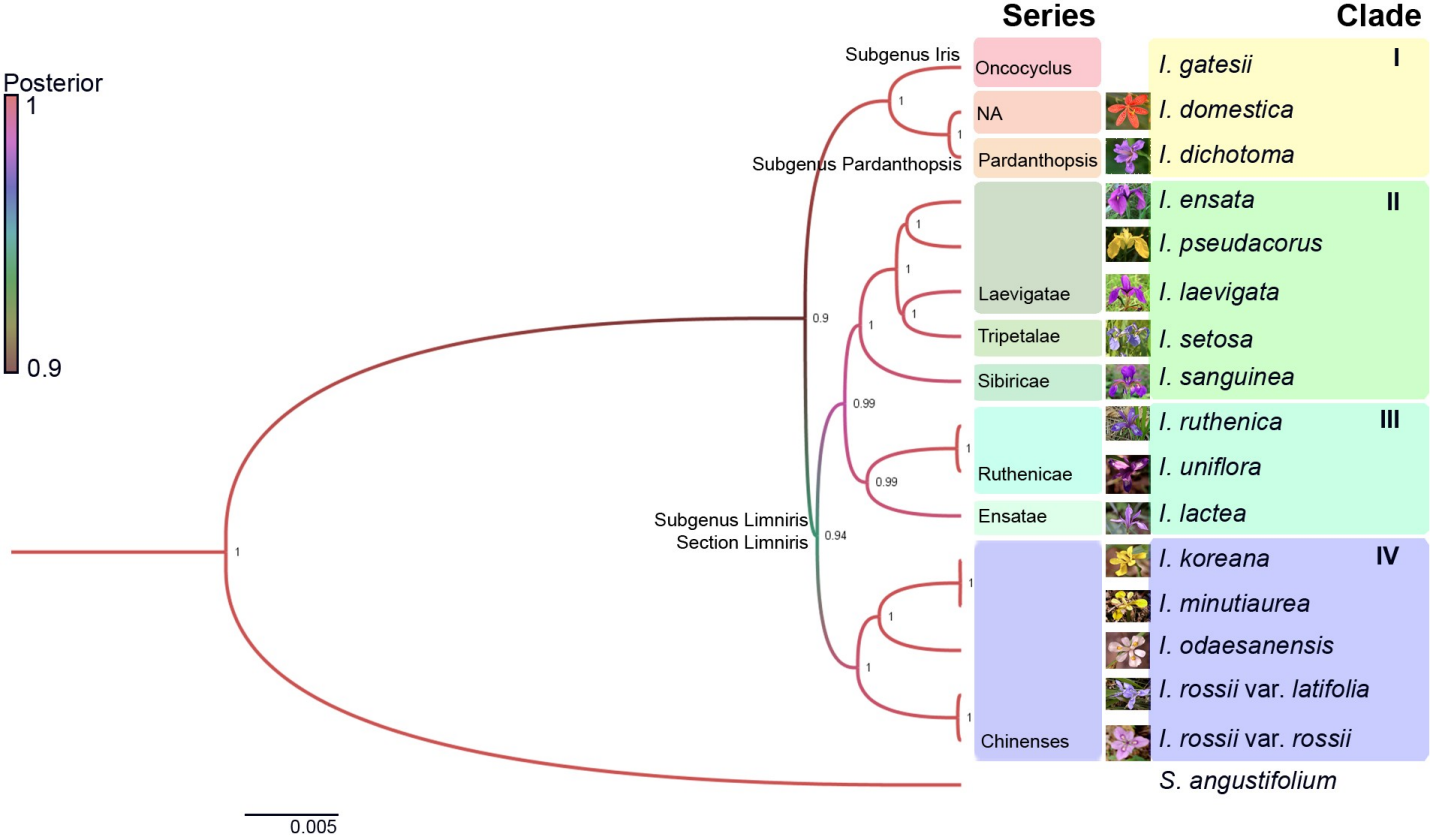
Bayesian inference phylogenetic tree generated using 57 intact single-copy plastid protein sequences that were predicted from each plastid genome assembly. The species with pictures are Korean-native *Iris*. The branch colours and values correspond to posterior values.

To determine a reliable pattern for *Iris* speciation, we implemented the Bayesian inference (BI) method with the BEAST software package [17]. Using 57 intact single-copy plastid genes that were predicted from each genome assembly, we built a species tree comprised of four distinct clades (Fig 2). The posterior probabilities on each branching node were within a reliable range, from 0.9 to 1, and all clades diverge from the outgroup, *S*. *angustifolium*. Clade I consists of *I*. *gatesii* (Subgenus *Iris*, Section *Oncocyclus*), together with *Iris domestica* and *Iris dichotoma*. Clades II, III, and IV represent the Subgenus *Limniris*. Clade II is comprised of *Iris ensata* (Subgenus *Limniris*, Section *Limniris*, Series *Laevigatae*), *Iris pseudacorus* (Subgenus *Limniris*, Section *Limniris*, Series *Laevigatae*), *I*. *setosa* (Subgenus *Limniris*, Section *Limniris*, Series *Tripetalae*), *I*. *laevigata* (Subgenus *Limniris*, Section *Limniris*, Series *Laevigatae*), and *I*. *sanguinea* (Subgenus *Limniris*, Section *Limniris*, Series *Sibiricae*). Clade III contains *I*. *ruthenica* (Subgenus *Limniris*, Section *Limniris*, Series *Ruthenicae*), *I*. *uniflora* (Subgenus *Limniris*, Section *Limniris*, Series *Ruthenicae*), and *Iris lactea* (Subgenus *Limniris*, Section *Limniris*, Series *Ensatae*). Clade IV in comprised of Series *Chinenses* and includes *I*. *koreana* (Subgenus *Limniris*, Section *Limniris*, Series *Chinenses*), *I*. *minutoaurea* (Subgenus *Limniris*, Section *Limniris*, Series *Chinenses*), *I*. *odaesanensis* (Subgenus *Limniris*, Section *Limniris*, Series *Chinenses*), and *I*. *rossii* (Subgenus *Limniris*, Section *Limniris*, Series *Chinenses*).

### Identification of plastid marker sequences to facilitate construction of phylogenetic trees

**Fig 3:**
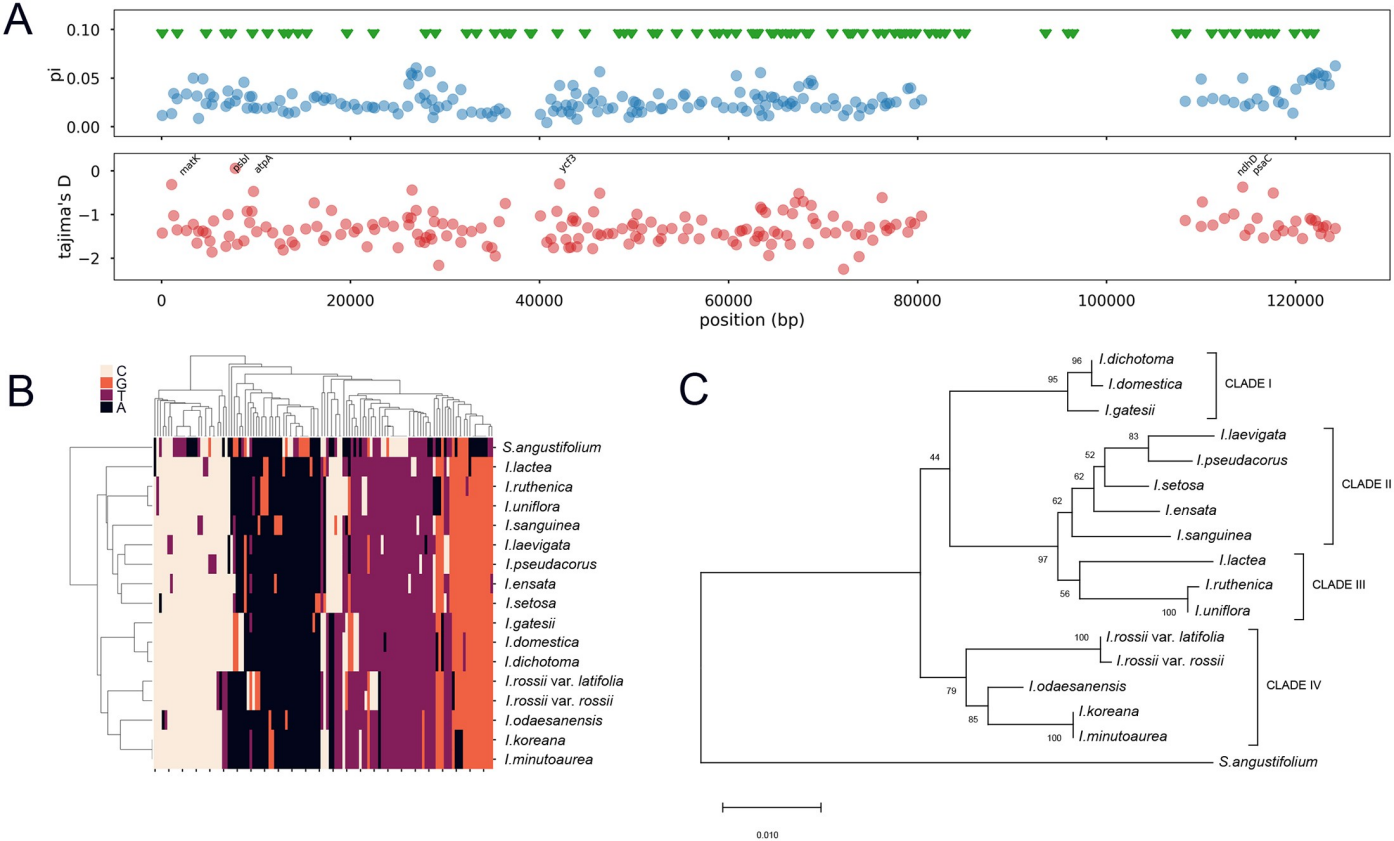
Plastid sub-genome selection by Tajima’s D statistics. (a) Whole-plastid genome Tajima’s D distribution revealing few regions with values higher than -0.5. Upper panel shows diversity (pi) values, and lower panel depicts Tajima’s D values. (b) Hierarchical clustering of samples (rows) and polymorphic sites (columns) in the selected plastid sub-genome matrix by Tajima’s D. (c) Maximum likelihood phylogenetic tree with 1,000 bootstrap values generated from the genotype matrix of the selected plastid sub-genomes by Tajima’s D.

Here, in order to select the sub-genomic regions for this analysis, we attempted to implement the Tajima’s D test that estimates the evolutional neutrality of observed genomic regions. Whole plastid genomes from our study were aligned using Cactus software [18]. Well-aligned sub-genomes were then collected, and both the diversity (pi) and Tajima’s D were calculated (Fig 3a). Theoretically, Tajima’s D can statistically detect a non-random evolution process, which includes various types of selection [19]. We detected five well-aligned sub-genomic regions showing Tajima’s D > -0.5 (Fig 3a). This threshold was determined at a higher bar than Tajima’s D = -0.9 from the *matK* sequence alignment of 117 Iris accessions (S2 Table). Our selected sub-genomic regions were 998 bp in total length (S3 Table), with 124 segregating sites on the alignments, excluding gaps. We further applied hierarchical clustering on the genotype matrix of segregating sites versus 17 species (Fig 3b). Notably, the dendrogram generated from our clustering analysis displayed consistent phylogeny with the BI species tree. The maximum likelihood (ML) tree with 1,000 bootstrap values on same genotype matrix also showed classification of Iris species consistent with the BI tree (Fig 3c), indicating that the 124 segregating sites we selected are informative enough to recapitulate the BI phylogenetic tree.

## Discussion

Using the whole-plastid genome assemblies of 14 native Korean *Iris* species determined in our study, together with the previously published *I*. *gatesii* and *I*. *sanguinea* plastid genomes [20, 21], we performed pairwise *Ks* calculation and BI phylogenetic tree construction with 57 non-redundant plastid genes to enable the observation of speciation among Korean-native *Iris*. From our BI phylogenetic tree, we observed that Series *Chinenses* species, which did not co-cluster in with *psbA-trnH* and *trnL-F*-based phylogenetic trees calculated in a previous study [7], formed a single clade in our analysis (Fig 2). In addition, *I*. *gatesii*, *I*. *dichotoma*, and *I*. *domestica* clustered into a single clade. Until recently, *I*. *domestica*, known as blackberry lily, has been considered to be a single species belonging to the genus *Belamcanda*, as *Belamcanda domestica*, due to its unique morphological features, such as subequal tepal and ligulate style branches, not found in most *Iris* species [22]. However, recent molecular studies using *matK* [22], *trnL-trnF*, and the plastid intergenic region [23] clearly showed that *I*. *domestica* is nested in the genus, *Iris*, and closely related to *I*. *dichotoma*. This species also has unique morphological characteristics, and as such, *I*. *dichotoma* has previously been classified in a separate subgenus, section, or subsection of the *Iris* genus, and, alternatively, has also been proposed as a member of the distinct genus *Pardanthopsis* [24–26]. In contrast, our BI tree showed that *I*. *domestica* and *I*. *dichotoma* are nested within the genus *Iris*, and are phylogenetically closely related, displaying both a short branch length (0.0014) and a BI posterior value of 1.0. Our plastid sequencing therefore shows a close phylogenetic relationship between *I*. *dichotoma* and *I*. *domestica* and supports the transfer of *Belamcanda domestica* into the *Iris* genus.

In order to construct a reliable species tree, it is important to select an informative genomic region that can classify query samples into the right clade. This can be accomplished using the entire set of single-copy genes; however, this is practically expensive with regards to the analysis procedures that are required. Based on the premise that our BI phylogenetic tree represents a reliable representation of phylogenetic relationships within the *Iris* genus, we then selected a subset of genomic regions that can recapitulate the BI phylogenetic tree topology with speciation signals calculated by Tajima’s D statistics. While Tajima’s D was originally hypothesized for estimating selective pressures within a single species [19], the set of genes showing notably high Tajima’s D value successfully recapped the topology of the BI phylogenetic tree. Nevertheless, our study still violates the original hypothesis of the Tajima’s D test, and it would be difficult to generalize the evolutionary neutral regions selected. Rather, we propose that the Tajima’s D distribution can capture the genomic regions preserving the speciation signals on the alignment blocks of highly conserved chloroplast sequences. Using the DNA barcode at the *matK* gene, our collection of 117 *Iris* samples showed a Tajima’s D value of -0.9. Using a slightly more conservative value (absolute Tajima’s D <0.5) as our threshold, we then calculated the Tajima’s D distribution in our whole *Iris* plastid genomes after multiple sequence alignment with the outgroup, *S*. *angustifolium*. Use of an outgroup introduces a number of rare alleles and increases the number of segregating sites in the alignments, causing the overall Tajima’s D distribution to shift towards positive selection (negative value), as compared to the Tajima’s D distribution without the outgroup. As expected, we observed that Tajima’s D values were distributed in the range lower than -1 (Fig 3). Only a few regions showed Tajima’s D values higher than our threshold, and these were selected as candidate plastid genome regions that may conserve *Iris* speciation signals. Notably, the phylogenetic tree constructed using concatenated sequences of the candidate representative regions successfully recapitulates the topology of the BI phylogenetic tree. Moreover, genes proximal to the candidate plastid genome representative regions include *matK*, *psbI*, *atpA*, *ycf3*, *ndhD*, and *psaC*. Interestingly, the *matK* and *psbI* regions, which have been used as noncoding spacers (psbK–psbI), have also been proposed as DNA barcode markers [27].

From our analysis, problematic species complexes were also confirmed using pairwise *Ks* values of the genes identified in plastid genomes. Pairwise *Ks* value distribution showed highly similar relationships between *I*. *koreana* vs. *I*. *minutoaurea* and *I*. *ruthenica* vs. *I*. *uniflora* (Fig 1b). These species are quite difficult to distinguish due to their lack of distinct morphological features, as well as the presence of suspected hybrids between these species [28, 29]. Here we found that, in addition to displaying low *Ks* values, these species did not form separate clades in the *matK*-based phylogenetic tree of 117 *Iris* accessions (S1 Fig). A previous phylogenetic study of Korean *Iris* species using partial plastid DNA sequences, such as psbA-trnH and trnL-F, also indicated that the phylogenetic relationship between *I*. *minutoaurea* and *I*. *koreana* was not clear and thus needed to be improved using diverse genetic markers to clarify ambiguous classification. Here, our results reveal that the delineation of those species complexes remains unclear and needs to be examined further.

In summary, we constructed the plastid genome assemblies of 14 Korean-native *Iris* species and performed Ks value-based classification. In addition, using a BI phylogeny calculated from the alignment of 57 predicted plastid proteins, we provide suggestions for resolving classification ambiguities within the *Iris* genus, and further identify representative plastid genomic regions that may be informative for cost-efficient classification. Critically, these findings provide a valuable resource for determining phylogenetic relationships within the *Iris* genus and can be further be utilised for the identification and protection of endangered *Iris* species.

## Methods

### Plant materials

Collection information for species used in this study is shown in Table 4. The voucher specimens are deposited in the herbaria of the Korean National Institute of Biological Resources (KB) and Daegu University (DGU). Young leaves were collected from plants, dried in silica gel, and store at -80°C until use.

**Table 4:** Species collected in this study.

Species	Notes	Collection Localities	Voucher Number
*S*. *angustifolium*		Korea, Jeju-do, Seogwipo	KB NIBRVP0000470408
*I*. *sanguinea*		Korea, Gyeonggi-do, Pocheon	KB NIBRVP0000138241
*I*. *laevigata*	Endangered	Korea, Gangwon-do, Goseong	KB NIBRVP0000472979
*I*. *setosa*		Korea, Gangwon-do, Goseong	KB NIBRVP0000410251
*I*. *ensata var*. *spontanea*		Korea, Gyeongsangbuk-do, Yeongdeok	KB NIBRVP0000281433
*I*. *pseudoacorus*		Korea, Jeollanam-do, Boseong	KB NIBRVP0000480267
*I*. *lactea var*. *chinensis*		Korea, Gyeongsangbuk-do, Yeongdeok	KB NIBRVP0000281459
*I*. *ruthenica*	Endangered	Korea, Daegu	DGU Won12627
*I*. *uniflora var*. *carcina*		Korea, Gangwon-do, Gangneung	KB NIBRVP0000612157
*I*. *domestica*		Korea, Incheon, National Institute of Biological Resources	Living collection
*I*. *dichotoma*		Korea, Incheon, Ongjin, Is. Baengyeong	KB NIBRGR0000107578
*I*. *odaesanensis*	Endemic to Korea	Korea, Gyeongsangbuk-do, Cheongsong	KB GPI2009-005C
*I*. *rosii var*. *latifolia*	Endemic to Korea	Korea, Gyeonggi-do, Yongin-si	KB NIBRVP0000480288
*I*. *rosii var*. *rosii*		Korea, Jeollanam-do, Haenam	KB NIBRVP0000480289
*I*. *koreana*	Endangered	Korea, Jeollanam-do, Jeongeup-si, Naejangsan National Park	KB GEIBVP0000170010
*I*. *minutoaurea*		Korea, Gyeonggi-do, Yangpyeong	KB NIBRGR0000164710

### DNA extraction and sequencing

Total DNA extraction from plant leaves was performed using the DNeasy Plant Mini Kit (QIAGEN, Hilden, Germany), and the HiGen Gel & PCR Purification Kit (Biofact Inc., Daejeon, Korea) was used for DNA purification. Extracted DNA was sequenced with the Illumina NGS platform and by the traditional Sanger sequencing method (Table 1). Sanger sequencing was performed as previously described [30]. Sequences of DNA fragments were determined using the ABI Prism BigDye Terminator Cycle Sequencing Kit, ver. 3.0 (QIAGEN) and an ABI 3700 Analyzer (Applied Biosystems, Foster City, CA) by genome walking methods. The chromatograms and alignments were visually checked and verified using Sequencer 5.0 (Gene Codes Corporation, Ann Arbor, MI, USA). Using Illumina NGS methods, about 4.4~6.9 million reads were generated on MiSeq platform for each *Iris* species. Around 3.5~5.5 million high quality reads obtained using quality_trim method (minimum quality score of 20) within the CLC assembly cell package, accounting for about 80% of raw reads and 0.9~1.5 Gb in length, were used for plastid genome assembly. Plastid genome-associated reads were extracted and reconstructed into full plastid genomes using CLC genome assembler, ver. 4.06 beta (CLC Inc, Rarhus, Denmark) software with manual inspection, yielding genomes ranging from 150 to 153 kb in length (Table 1). Assembled plastid genomes were annotated for genes, rRNAs, and tRNAs with GeSeq [31] and tRNAscan-SE software [32]. The annotations were curated to meet NCBI submission criteria, as follows: 1) plastid genome sequences showing internal stop codons were changed into ‘N’ and 2) genes missing the start and/or stop codon were removed.

### Bayesian tree construction

Genes predicted in plastid genomes were filtered using the following criteria: 1) they must be present in only one copy and 2) they must not contain any ‘N’s. Using these criteria, a total of 57 plastid genes was extracted from each of the 17 *Iris* species. The protein sequences encoded by these genes were then aligned using PRANK software [33], and protein alignments of the 57 gene products were parsed using BEAUti software. Construction of the Bayesian species tree was performed with BEAST software, ver. 1.10.4 [17], and this process was initiated with a random starting tree. Two runs of the Markov Chain Monte Carlo (MCMC) chain, at 50 million generations were implemented, with sampling at every 5,000 steps. The relaxed-clock model was used with lognormally distributed uncorrelated rates. To assign the protein evolutionary model, we used ProtTest software for the alignments and selected the best model with PlastidREV [34].

### Phylogenetic analysis

To estimate the selection process that occurred for each gene in the plastid genome, Tajima’s D statistics were applied using DendroPy [35]. The coding sequences of our 57 unique single-copy plastid genes were extracted and aligned using PRANK, with the option, ‘-codon’ [33]. The resulting FASTA alignment files were supplied to the dendropy.calculate.popgenstat.tajimas_d module, with ignore_uncertain = True. Pairwise comparisons were performed using PRANK software, and the *Ks* values were calculated with KaKs_Calculator [36]. The phylogenetic tree generated from selected plastid genome regions was inferred by the maximum likelihood method and the Tamura-Nei model [37], and these analyses were conducted with the MEGA X software package [38].

## Supporting information

S1 Fig Phylogenetic tree of the 117 Iris collection by the DNA barcode at the matK gene. (PDF)

S1 Table 83 predicted genes in each assembled CP genome (this includes incomplete genes). (XLSX)

S2 Table Segregating site of matK region from 117 Iris species. (XLSX)

S3 Table CP sub genomes with less selection pressure. (XLSX)
