# Supplementary figures and images for: Identification of plastid genomic regions inferring species identity from de novo plastid genome assembly of 14 Korean-native Iris species (Iridaceae)
Source: PLoS One. 2020 Oct 26;15(10):e0241178. doi: 10.1371/journal.pone.0241178 (PMC7588056; doi:10.1371/journal.pone.0241178)

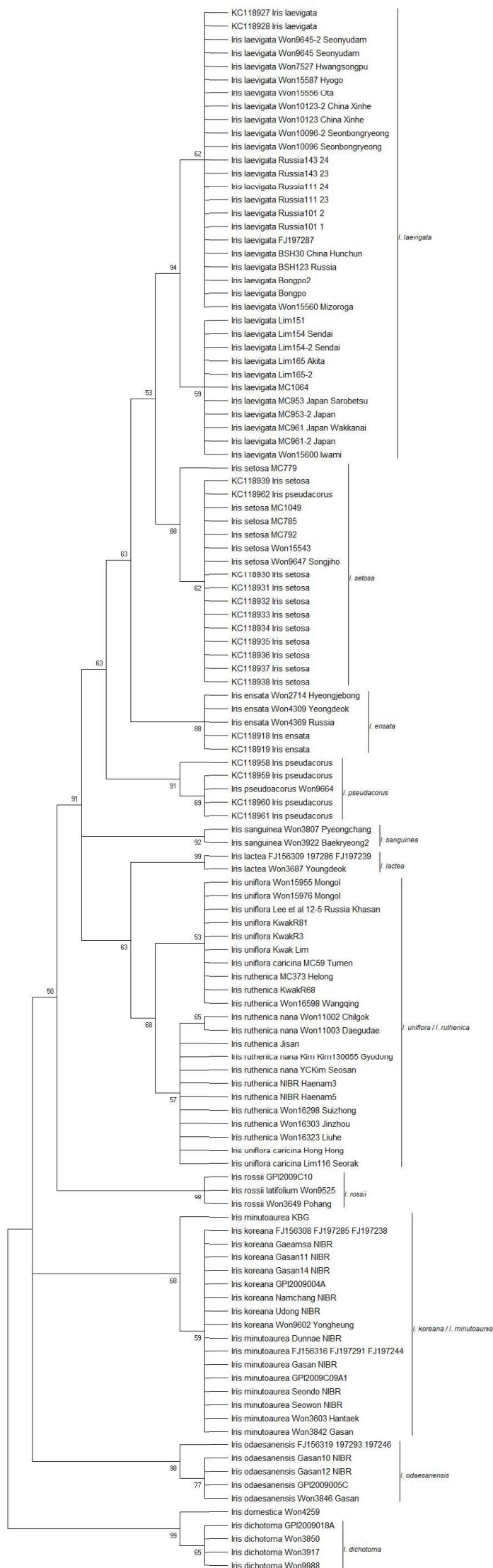

Supplement: S1 Fig — (PDF) [file pone.0241178.s001.pdf]
